# Clinical Practice Patterns of Assessment and Interventions for Elderly Patients with a Hip Fracture Who Are at Risk of Dysphagia—A Survey

**DOI:** 10.3390/diseases13080253

**Published:** 2025-08-08

**Authors:** Stine Mølgaard Kristoffersen, Signe Westmark, Dorte Melgaard

**Affiliations:** 1 Department of Physiotherapy and Occupational Therapy, Horsens Regional Hospital, 8700 Horsens, Denmark; stimoa@rm.dk; 2 Center for Clinical Research, North Denmark Regional Hospital, 9800 Hjoerring, Denmark; signe@westmark.dk; 3 Department of Clinical Medicine, Aalborg University, 9000 Aalborg, Denmark; 4 EMRUn, Department of Acute Medicine and Trauma Care, Aalborg University Hospital, Hobrovej 18-22, 9000 Aalborg, Denmark

**Keywords:** hip fracture surgery, assessment, treatment, intervention, swallowing disorders, old age

## Abstract

Objective: Dysphagia is common among elderly patients after hip fracture surgery and can lead to aspiration pneumonia, malnutrition, and delayed rehabilitation. This study aims to present current clinical practice patterns of assessment and intervention for dysphagia in this patient group. Methods: The study was conducted through a two-round online questionnaire targeting Danish occupational therapists with expertise in dysphagia post hip fracture. Results: A total of 71 therapists participated in round one, and 44 (62%) completed round two. Triggers for assessment included coughing, recurrent pneumonia, voice changes, altered eating habits, unplanned weight loss, functional decline, and comorbidities; age was rarely used. Frequently used assessment tools were Facio-Oral Tract Therapy (57.1%), the Minimal Eating Observation Form—Version II (40%) and the Volume–Viscosity Swallow Test (41.4%). Key interventions included texture modification, posture correction, patient education, oral hygiene optimization, compensatory strategies, and dysphagia training; oral screens and electrical stimulation were less common. Conclusions: This study provides a descriptive overview of current dysphagia assessment triggers, tools, and interventions used for elderly hip fracture patients in Denmark. The findings highlight clinical practice patterns that can inform future research on patient outcomes and the effectiveness of specific interventions in this population.

## 1. Introduction

Eating and drinking are essential for daily life and survival. When these activities become difficult, they can negatively affect functional ability, quality of life (QoL), and social relationships [1,2]. Swallowing difficulties, also known as dysphagia, may occur as a result of mechanical obstruction, causing problems in meeting the basic needs of eating and drinking [3]. Dysphagia is related to factors such as age, comorbidity, poor dental status, and cognition [1,4]. Symptoms include impaired bolus formation and movement, leading to issues like aspiration, coughing during meals, difficulty swallowing saliva, or frequent swallowing [1].

These risks are particularly pronounced among older adults, who are more vulnerable to dysphagia-related complications such as malnutrition, aspiration pneumonia, involuntary weight loss, functional decline, hospital readmission, and even higher mortality [1,5,6]. In addition, malnutrition and functional decline have been associated with increased fall risk, delayed recovery, and reduced ability to perform activities of daily living after discharge [7,8,9,10].

With an aging population, the incidence of falls and related injuries is rising, and global hip fracture cases are projected to reach 4.5 million by 2050 [11]. Older patients undergoing hip fracture surgery are particularly affected, with reported dysphagia prevalence ranging from 5% to 77% [12,13,14,15,16,17,18,19]. Despite this high prevalence, there is no consensus on optimal screening tools or intervention strategies, and clinical practice varies considerably. This reflects both differences in available resources and the inherent complexity of dysphagia management in older adults, where some approaches are simple to implement, while others require specialist expertise [20].

Despite the severe consequences of dysphagia in this population, no comprehensive guidelines exist for systematic assessment and management in elderly hip fracture patients [1,6,15,21,22]. Meeting the needs of older patients with dysphagia is a multifaceted process, as recommendations vary in complexity and adherence is inconsistent, highlighting the importance of matching interventions to individual needs [20].

To address this lack of consensus, a clearer understanding of current clinical practices is needed. Accordingly, this study aimed to answer the following questions:

1. What is the level of self-reported experience among dysphagia therapists (DTs) in Denmark?
2. What key parameters are used to initiate and conduct dysphagia assessments in patients with hip fractures?
3. What are the most common assessment tools used for dysphagia in patients with hip fractures?
4. What parameters trigger the need for further dysphagia assessment in patients with hip fractures?
5. Which dysphagia interventions are most commonly reported by therapists for patients with hip fractures?

By addressing these questions, this study describes current clinical practices and identifies patterns that may inform future research and guideline development for managing dysphagia in elderly hip fracture patients.

## 2. Materials and Methods

This survey study aimed to explore clinical practices related to dysphagia assessment and intervention in patients undergoing hip fracture surgery. Specific to this study, the survey can provide valuable knowledge for assessment and intervention among patients with dysphagia who have undergone hip fracture surgery.

To develop the questionnaires, a literature review was conducted and combined with clinical knowledge. This process informed the selection of assessment tools and interventions for patients at risk of dysphagia. The online questionnaires were piloted, and minor adjustments were made based on feedback. Two survey rounds were completed between December 2020 and February 2021. Data were collected and managed using Research Electronic Data Capture (REDCap), hosted by the North Denmark Region [23,24]. REDCap is a secure, web-based software platform designed to support data capture in research studies.

In Denmark, dysphagia therapists (DTs) with a background in occupational therapy are responsible for assessing and treating dysphagia in collaboration with a multidisciplinary team, including doctors, nurses, and dietitians. The inclusion criteria for this study required DTs to have experience in assessing and/or treating patients undergoing hip fracture surgery who are at risk of dysphagia. The initial screening question ensured that only those with experience in dysphagia assessment or treatment participated. The participants included in round one were therefore not individually selected by the authors, but represented a self-selected group recruited openly through the Danish Society of Swallowing Disorders, the Occupational Therapy Society for Dysphagia, the Occupational Therapy Society for Research, and the authors’ professional networks. Recruitment was followed by a snowballing method, where participants and professionals within the authors’ networks were asked to refer other potential participants [25].

Before the first round, participants provided informed consent based on detailed written study information, including how their responses would be stored and handled, assurances of anonymity, and the option to withdraw at any time. Consent was obtained by participants answering a yes/no question confirming their willingness to participate under these conditions. Participants who did not wish to participate could simply close the survey link, and no data would be recorded from them. They then answered questions about their experience with dysphagia, patients with hip fractures, and details about their primary workplace.

The goal of the first round was to gain insights into the tools, considerations, and key parameters that participants used in assessing and treating dysphagia in patients undergoing hip fracture surgery. Participants responded to non-standardized questions on (1) reasons for initiating dysphagia assessments, (2) the assessment tools they used, and (3) the extent to which they applied specific interventions. To capture detailed clinical practices, participants were asked about the standardized or modified use of these tools and how frequently they employed them. Free text options were available for additional comments if the provided categories were insufficient. The information from this round was used to develop the questions for the second round.

The second round aimed to gather more detailed information about the participants’ clinical practices. A 5-point Likert scale was primarily used to measure the participants’ level of agreement [26,27]. See Tables S1 and S2 for the full questionnaire from round one and round two.

Demographic data from the first round were collected, categorized, and presented as numbers and percentages. The set-up in REDCap ensured that questions were fulfilled before completion. All analyses were performed using R version 3.5.3 (R Core Team, Vienna, Austria).

The study was conducted according to the Declaration of Helsinki and informed consent was obtained electronically from all participants. The collected data were anonymised. This study was approved by the Danish Data Protection Agency (K2022-039). According to Danish legislation, this type of study does not need ethical approval.

## 3. Results

Two rounds of a survey were performed, with 71 DTs from the five regions in Denmark participating in round one, and 44 of the 71 (62%) participants completing the second round. The majority of the participating DTs had 6 to 24 months of experience working with patients suffering from dysphagia after undergoing hip fracture surgery (Table 1).

**Figure 1:**
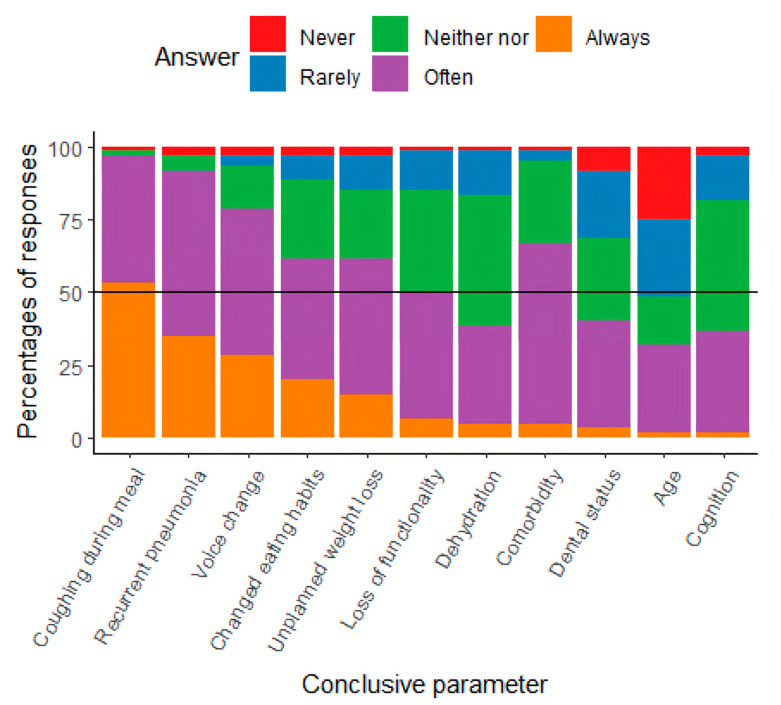
Results from the study, presented with the percentage of responses about conclusive parameters for initiating assessment.

Participants identified various key parameters for initiating and conducting dysphagia assessments. They reported that triggers for assessment were either noted in the patients’ medical records or based on observations from colleagues. As shown in Figure 1, common parameters for initiating assessments included coughing, recurrent pneumonia, voice changes, altered eating habits, unplanned weight loss, loss of function, and comorbidities, which were frequently or always considered important. Age was rarely used as a trigger for initiating dysphagia assessments.

The three most common assessment tools presented by the participants in this study were Facio-Oral Tract Therapy (F.O.T.T.), the MEOF-II, and the V-VST (see Table 2). These three tools were reported to be easy and quick to use. Participants using F.O.T.T. and/or the MEOF-II indicated that they were adequate for uncovering dysphagia. Among participants who used the V-VST, there was an equal distribution of those who indicated that the tool was adequate and those who reported the need for further assessment.

**Figure 2:**
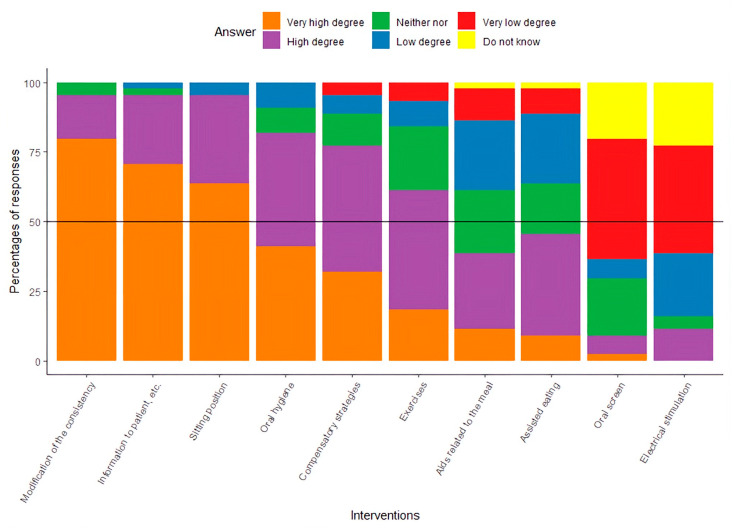
Results presented with the percentage of responses about the clinical use of specified interventions.

As presented in Figure 2, 6 out of 10 of the intervention possibilities were in the group of ‘High degree’ or ‘Very high degree’: modification of the consistency of the food, informing patients about dysphagia, correction of the patient’s sitting position, optimization of oral hygiene, instruction in compensatory strategies, and specific training related to dysphagia. With regard to the use of an oral screen and electrical stimulation as a part of the intervention, participants’ responses indicated a ‘Low degree’ or ‘Very low degree’ of clinical use.

## 4. Discussion

This study aimed to outline clinical practices regarding the key parameters that should trigger dysphagia assessments in elderly patients with hip fractures, the tools used for assessment, and the interventions applied.

In summary, the findings of this study directly address the five predefined research aims:

1. Self-reported experience of DTs: Most participating dysphagia therapists had between 6 and 24 months of experience with hip fracture patients, indicating a relatively recent but emerging focus in practice.
2. Key parameters for assessment initiation: Clinical indicators such as coughing, recurrent pneumonia, voice changes, altered eating habits, weight loss, functional decline, and comorbidity were most frequently used, while age alone was rarely applied.
3. Assessment tools used: F.O.T.T., MEOF-II, and V-VST were the most frequently used tools, with F.O.T.T. being more prominent in Danish practice compared to international trends.
4. Triggers for further assessment: Inadequate findings from initial screening often led to more detailed evaluation, though instrumental methods (FEES/VFS) were rarely accessible.
5. Common interventions applied: Therapists most frequently reported food texture modification, posture correction, oral hygiene, patient education, compensatory strategies, and dysphagia-related training—though the efficacy of these interventions in hip fracture patients remains undocumented.

Collectively, these findings describe current practice patterns and identify both overlaps with general geriatric dysphagia care and hip fracture-specific challenges that warrant targeted research on patient outcomes and effective interventions. The participants identified coughing during meals and general voice changes as important conclusive parameters, which are supported by the literature as symptoms of dysphagia [13,19]. The literature documents that recurrent pneumonia and postoperative pneumonia among elderly patients, in general, may be a sign of dysphagia [1,28,29], and this was confirmed by participants in this study. Identifying and treating dysphagia is a multidisciplinary effort [30,31], and the results of this study indicate that colleagues’ observations are an important conclusive parameter for initiating the assessment of dysphagia. One study points out that when elderly patients with hip fracture experience problems related to eating and drinking, they may change their eating habits or lose weight [13], leading to an increased risk of malnutrition, impaired functionality, hospital readmission, impaired QoL, and higher mortality. These results are supported by studies including geriatric patients [1,5,30,31]. The participants in the survey indicated that they ‘Never’ or ‘Rarely’ used age as a conclusive parameter when initiating an assessment of dysphagia. Advanced age is generally highlighted in the literature as a common feature in dysphagia [1,15,17,18,19,31]. Instead of merely proposing additional tools for the identification of dysphagia in hip fracture patients, our findings point towards the need for increased awareness among healthcare professionals of the elevated risk of dysphagia in this population. Dysphagia has been classified as a geriatric syndrome [1], and several studies highlight that elderly people are susceptible to dysphagia because the normal aging process and impaired functionality, when combined with stressors such as hip fracture, increase this risk [11,14,24]. Raising awareness of these risk factors may be as crucial as implementing formal screening tools. Targeted education for staff could ensure that subtle signs—such as coughing during meals, voice changes, or unintentional weight loss—are recognized early and prompt timely assessment. Therefore, improving awareness and clinical vigilance regarding dysphagia in hip fracture patients should be considered an important first step, complementing any future implementation of additional screening tools. No assessment tools for dysphagia in elderly patients with hip fractures have been developed and validated, to the authors knowledge. In the literature, a non-specific assessment for dysphagia is often used, while some other studies use the V-VST or/and the MEOF-II, both of which are validated for the geriatric population [32,33]. These two tools were also highlighted by the participants in our survey, though the participants primarily used the F.O.T.T., which is used in more clinical contexts in Denmark. The gold-standard assessment for dysphagia is videofluoroscopy (VFS) and Fiberoptic Endoscopic Evaluation of Swallowing (FEES), which can diagnose aspiration and other physiological problems in the oral phase and its subsequent impact in the pharyngeal phase [34]. None of the studies included in this study used these instrumental assessments, which may be due to limited access to them, but also due to the fact that more of the elderly may be challenged in terms of participating in an instrumental assessment. In Denmark, FEES is typically available, but primarily for neurological patients, while access to VFSS is limited and mainly offered to patients with head and neck cancer.

To the authors’ knowledge, no studies have documented the effects of interventions targeting dysphagia in elderly patients with hip fractures. The participants in this study follow a clinical practice that includes the following interventions: modifying food consistency, providing patient education on dysphagia, correcting sitting posture, improving oral hygiene, teaching compensatory strategies, and providing specific dysphagia-related training. As noted, elderly patients with hip fractures share many characteristics with geriatric patients, making it relevant to draw from existing knowledge on treatment in that group. There is evidence that modifying food consistency can reduce the risk of aspiration; however, it may also increase the risk of dehydration, malnutrition, and a lower quality of life [35].

Patient education is generally valued, including by those with oropharyngeal dysphagia (OD); however, no studies have documented the effects of providing dysphagia-related information to elderly patients with hip fractures [36]. Patients with hip fractures are very challenged in terms of their sitting position [15]; one study documented some effects of correcting the sitting position of nursing home residents on reducing the risk of aspiration pneumonia [37]. More studies document the effect of optimizing oral hygiene on reducing the risk of aspiration pneumonia [38,39]. There is limited documentation of the effect of compensatory strategies and specific training related to dysphagia in the elderly [40].

The number of participants included in both rounds of the questionnaire is a strength of this study, as it falls within the recommended range of 10 to 30 experts that is typically considered sufficient to ensure a reliable consensus in this methodology [27,41]. The response rate for round two of 62% is considered acceptable. Participants were recruited through professional networks and voluntary self-enrollment, representing both the primary and secondary healthcare sectors. However, as there is currently no national registry of DTs in Denmark, it was not possible to determine the proportion of the total DT population captured in this study. This recruitment strategy may therefore introduce selection bias, as those who chose to participate might differ systematically from non-participants (e.g., in motivation, experience, or interest in the topic). Such potential bias is a well-recognized limitation of survey-based research, and should be considered when interpreting the generalizability of the findings.

This study provides novel, hip fracture-specific data on dysphagia practices in Denmark. While many of the assessment tools and interventions reported mirror those used in broader geriatric populations [1], our findings highlight two points of distinction. First, age alone was rarely used as a trigger for assessment, despite its recognition as a general dysphagia risk factor in geriatric care. Second, the frequent use of F.O.T.T. reflects a practice pattern that is less commonly reported internationally, where tools such as the V-VST and MEOF-II predominate. These results emphasize that while clinical approaches overlap with those used for geriatric patients in general, hip fracture patients represent a subgroup characterized by acute postoperative vulnerability, reduced mobility, and high dysphagia prevalence, underscoring the need for tailored awareness and further research.

## 5. Conclusions

This study is the first to describe current dysphagia assessment and intervention practices among therapists working with elderly hip fracture patients. The findings show the following

- The V-VST, F.O.T.T., and the MEOF-II were the most frequently reported assessment tools, suggesting their widespread use in Danish practice.
- Clinicians reported commonly using interventions such as food consistency modification, patient education, posture correction, oral hygiene optimization, compensatory strategies, and dysphagia-related training; however, the effectiveness of these approaches was not evaluated in this study.

In conclusion, dysphagia practices for hip fracture patients appear to largely mirror those used in other elderly populations. This study does not evaluate patient outcomes or the effectiveness of specific assessments or interventions, but provides novel, hip fracture-specific data to inform future research and the development of evidence-based, multi-professional guidelines. Additionally, more research is needed to explore patient perspectives following dysphagia assessment.

## Figures and Tables

**Table 1:** Demographics of participants.

		Round 1 *n* = 70 (%)	Round 2 *n* = 44 (%)
Level of education	Basic	63 (90.0)	38 (86.4)
	Master	5 (7.1)	4 (9.1)
	PhD	0	0
	Other	2 (2.9)	2 (4.5)
Work engagement related to dysphagia	Clinical	66 (94.3)	41 (93.2)
	Academic	0	0
	Equally clinical and academic	4 (5.7)	3 (6.8)
Primary workplace	Hospital	23 (32.9)	16 (36.4)
	Primary healthcare sector (municipality)	46 (65.7)	27 (61.4)
	Private	1 (1.4)	1 (2.3)
Region	The Capital Region of Denmark	11 (15.7)	5 (11.4)
	Central Denmark Region	23 (32.9)	15 (34.1)
	North Denmark Region	19 (27.1)	13 (29.5)
	Region Zealand	8 (11.4)	6 (13.6)
	Region of Southern Denmark	8 (11.4)	5 (11.4)
	Abroad	1 (1.4)	0
Experience working with patients with dysphagia and operated on for hip fracture	None	4 (5.7)	0
	<½–2 years	27 (38.5)	17 (38.6)
	2–8 years	28 (40.1)	20 (45.5)
	>8 years	11 (15.7)	7 (15.9)

**Table 2:** Most commonly used assessment tools.

Assessment Tool		F.O.T.T.	FEES	GUESS	MEOF-II	EAT-10	MISA	V-VST	Water Test	Other ***
Use of the tool *		40 (57.1)	0	5 (7.1)	28 (40.0)	15 (21.4)	17 (24.3)	29 (41.4)	20 (28.6)	4 (5.7)
Experience of the tool **	Easy to use	18 (25.7)		1 (1.4)	21 (30.0)	9 (12.9)	5 (7.1)	17 (24.3)	14 (20.0)	4 (5.7)
	Quick to use	8 (11.4)		2 (2.9)	6 (8.6)	8 (11.4)	3 (4.3)	11 (15.7)	8 (11.4)	3 (4.3)
	Difficult to use	5 (7.1)		2 (2.9)	2 (2.9)	0	5 (7.1)	2 (2.9)	0	0
	Takes a long time	13 (18.6)		0	4 (5.7)	0	8 (11.4)	3 (4.3)	1 (1.4)	0
The results of the tool	It covers what is needed	20 (50.0)		1 (20.0)	15 (53.6)	5 (33.3)	10 (58.8)	14 (48.3)	6 (30.0)	1 (25.0)
	There is often a need for further assessments	15 (37.5)		4 (80.0)	13 (46.4)	10 (66.7)	5 (29.4)	14 (48.3)	14 (70.0)	2 (50.0)
	My colleagues have difficulty applying the results of the assessment	1 (2.5)		0	0	0	0	0	0	0
Time used compared to knowledge gained	Very good	7 (17.5)		0	10 (35.7)	3 (20.0)	8 (47.1)	11 (37.9)	7 (35.0)	2 (50.0)
	Good	25 (62.5)		3 (60.0)	17 (60.7)	8 (53.3)	5 (29.4)	16 (55.2)	9 (45.0)	2 (50.0)
	Neither nor	7 (17.5)		2 (40)	1 (3.6)	4 (26.7)	1 (5.9)	1 (3.4)	4 (20.0)	0
	Bad	1 (2.5)		0	0	0	3 (17.6)	1 (3.4)	0	0
	Very bad	0		0	0	0	0	0	0	0

Facio-Oral Tract Therapy (F.O.T.T.), Fiberoptic Endoscopic Evaluation of Swallowing (FEES), Gugging Swallowing Screen (GUSS), Minimal Eating Observation Form—Version II (MEOF-II), The Eating Assessment Tool (EAT-10), The McGill Ingestive Skills Assessment (MISA), The Volume–Viscosity Swallow Test (V-VST).* Participants could choose all the tools they were using.** Participants could choose up to two statements. *** Yale swallow protocol, oral placement therapy, modified barium swallow, Facial-Oral Tract Therapy Swallowing Assessment of Saliva (FOTT-SAS).

## Data Availability

The data presented in this study are available on request from the corresponding author.

## Supplementary Materials

The following supporting information can be downloaded at: https://www.mdpi.com/article/10.3390/diseases13080253/s1, Table S1: Questionnaire Round 1; Table S2: Questionnaire Round 2.

## Abbreviations

The following abbreviations are used in this manuscript:

QoL	Quality of life
DTs	Dysphagia therapists
REDCap	Research Electronic Data Capture
F.O.T.T.	Facio-Oral Tract Therapy
FEES	Fiberoptic Endoscopic Evaluation of Swallowing
GUSS	Gugging Swallowing Screen
MEOF-II	Minimal Eating Observation Form—Version II
EAT-10	The Eating Assessment Tool
MISA	The McGill Ingestive Skills Assessment
V-VST	The Volume–Viscosity Swallow Test
FOTT-SAS	Facial-Oral Tract Therapy Swallowing Assessment of Saliva
VFS	Videofluoroscopy
OD	Oropharyngeal dysphagia
