# Supplementary material for: Clinical Practice Patterns of Assessment and Interventions for Elderly Patients with a Hip Fracture Who Are at Risk of Dysphagia—A Survey
Source: Diseases. 2025 Aug 8;13(8):253. doi: 10.3390/diseases13080253 (PMC12385465; doi:10.3390/diseases13080253)
Supplement: Supplementary file 1 [file diseases-13-00253-s001.zip › diseases-3782235-supplementary.pdf]

# Supplementary Materials - Questionnaire

Information about informed content and demographic is not a part of this appendix.

## Table S1：Questionnaire Round 1

| Number | Question | Response option |
|---|---|---|
| 1 | When a patient with a hip fracture needs to be examined for dysphagia, what are the decisive parameters for you to initiate an examination? (Indicate the 5 you find most relevant) | - Dehydration<br>- Poor dental status<br>- Repeated unexplained pneumonias<br>- Coughing during meals or when swallowing saliva<br>- Observations from interdisciplinary colleagues<br>- The patient’s age<br>- The patient’s cognitive function<br>- Weight loss<br>- Changed functional ability<br>- Changed eating habits<br>- Changed voice<br>- Other (Specify) |
| 2 | Which dysphagia screening or examination tools do you use in elderly patients with hip fractures? | - Blue dye<br>- Facio Oral Tract Therapy (F.O.T.T.)<br>- Flexible Endoscopic Evaluation of Swallowing (FEES)<br>- Gugging Swallowing Screen (GUSS)<br>- Minimal Eating Observation Form – version II (MEOF-II)<br>- The McGill Ingestive Skills Assessment (MISA)<br>- The Volume-Viscosity Swallow Test (V-VST)<br>- Water test<br>- Other (Specify) |

| | | |
|---|---|---|
| 3 | How often do you use the tool? | The tools checked in question 2 will appear here with the following options:<br>- Used daily<br>- Used weekly<br>- Used monthly<br>- Used less frequently than monthly |
| 4 | What are your experiences with the tool? | The tools from question 2 will appear here. Two options can be checked:<br><br>- It is easy to carry out<br>- It is quick to carry out<br>- It is difficult to carry out<br>- It is time-consuming |
| 5 | How useful is the tool? | The tools from question 2 will appear here.<br><br>- It covers what is needed<br>- Often requires further examinations when I use the tool<br>- My colleagues have difficulty using the results from the examination with the tool<br>- Other (Specify) |
| 6 | How does your time consumption correspond with the knowledge the tool provides? | The tools from question 2 will appear here.<br>- Very good<br>- Good<br>- Neutral<br>- Poor<br>- Very poor |
| 7 | Which interventions do you most often initiate for patients with dysphagia and hip fracture? | - Oral hygiene recommendations<br>- Texture modifications – both food and liquid |

| | | |
|---|---|---|
| | | - Initiating training exercises based on results from my examination<br>- Initiating therapeutic feeding<br>- Initiating compensatory strategies during meals<br>- Adjustment of sitting position<br>- Provision of assistive devices for eating and drinking<br>- Other (Specify) |
| 8 | How often do you use the initiated intervention? | The interventions marked as used will appear here with the following options:<br>- Used daily<br>- Used weekly<br>- Used monthly<br>- Used less frequently than monthly |

## Table S2：Questionnaire Round 2

| Number | Question | Response option |
|---|---|---|
| 1 | When you encounter patients with hip fracture and suspected dysphagia, how often are the following parameters the reason you initiate a dysphagia examination?<br>- Age<br>- Underlying disease<br>- Dehydration<br>- Poor dental status<br>- Repeated unexplained pneumonias<br>- Coughing during meals or when swallowing saliva | Response options for each cause:<br>- Never the reason<br>- Rarely the reason<br>- Neutral<br>- Often the reason<br>- Always the reason<br>- I am not familiar with it |

| | | |
|---|---|---|
| | - Cognitive function<br>- Observations from interdisciplinary colleagues<br>- Weight loss<br>- Changed functional ability<br>- Changed eating habits<br>- Changed voice | |
| 2 | When you encounter patients with hip fracture and dysphagia in your practice, to what extent do you use the following interventions?<br>- Oral hygiene recommendations<br>- Electrical stimulation<br>- Texture modifications – both food and liquid<br>- Training exercises based on examination results<br>- Therapeutic feeding<br>- Compensatory strategies during meals<br>- Mouth guard / oral trainer<br>- Adjustment of sitting position<br>- Provision of assistive devices for eating and drinking<br>- Guidance of patient, relatives, and/or staff | Response options for each intervention:<br>- To a very low degree<br>- To a low degree<br>- Neutral<br>- To a high degree<br>- To a very high degree<br>- No experience |
